# Dietary Macronutrient Intake May Influence the Effects of TCF7L2 rs7901695 Genetic Variants on Glucose Homeostasis and Obesity-Related Parameters: A Cross-Sectional Population-Based Study

**DOI:** 10.3390/nu13061936

**Published:** 2021-06-04

**Authors:** Witold Bauer, Edyta Adamska-Patruno, Urszula Krasowska, Monika Moroz, Joanna Fiedorczuk, Przemyslaw Czajkowski, Dorota Bielska, Maria Gorska, Adam Kretowski

**Affiliations:** 1 Clinical Research Centre, Medical University of Bialystok, Marii Sklodowskiej-Curie 24A, 15-276 Bialystok, Poland; witold.bauer@umb.edu.pl (W.B.); urszula.krasowska@umb.edu.pl (U.K.); przemyslaw.czajkowski@umb.edu.pl (P.C.); adamkretowski@wp.pl (A.K.); 2 Clinical Research Centre, Medical University of Bialystok Clinical Hospital, Marii Sklodowskiej-Curie 24A, 15-276 Bialystok, Poland; monika_bakun@wp.pl (M.M.); j.fiedorczuk@wp.pl (J.F.); 3 Department of Family Medicine, Medical University of Bialystok, Mieszka I 4b, 15-054 Białystok, Poland; d.bielska1@wp.pl; 4 Department of Endocrinology, Diabetology and Internal Medicine, Medical University of Bialystok, Marii Sklodowskiej-Curie 24A, 15-276 Bialystok, Poland; mgorska25@wp.pl

**Keywords:** macronutrient intake, obesity, body fat content, glucose homeostasis, type 2 diabetes risk, TCF7L2 gene, rs7901695 single nucleotide polymorphism

## Abstract

Transcription factor-7–like 2 (TCF7L2) is one of the most important susceptibility genes for type 2 diabetes mellitus (T2DM). The aim of our cross-sectional population-based study was to analyze whether daily macronutrient intake may influence the effects of the TCF7L2 rs7901695 genotype on glucose homeostasis and obesity-related parameters. We recruited 810 participants (47.5% men and 52.5% women), 18–79 years old (mean age, 42.1 (±14.5) years), who were genotyped for the common TCF7L2 rs7901695 single-nucleotide polymorphism (SNP), and anthropometric measurements, body composition, body fat distribution (visceral (VAT) and subcutaneous adipose tissue (SAT) content), blood glucose and insulin concentrations after fasting and during OGTTs, and HbA1c were assessed. The VAT/SAT ratio, HOMA-IR (homeostatic model assessment of insulin resistance), HOMA-B (homeostatic model assessment of β-cell function), and CIR30 (corrected insulin response) were calculated. The daily macronutrient intake was evaluated based on 3-day food-intake diaries. Daily physical activity was evaluated based on a validated questionnaire. We performed ANOVA or Kruskal–Wallis tests, and multivariate linear regression models were created to evaluate the effects of dietary macronutrient intake on glucose homeostasis and obesity-related parameters in carriers of the investigated genotypes. This study was registered at ClinicalTrials.gov as NCT03792685. The TT-genotype carriers stratified to the upper protein intake quantiles presented higher HbA1c levels than the CT- and CC-genotype participants in the same quantiles (*p* = 0.038 and *p* = 0.022, respectively). Moreover, we observed higher HOMA-IR (*p* = 0.014), as well as significantly higher blood glucose and insulin concentrations, during the OGTTs for those in the upper quantiles, when compared to subjects from the lower quantiles of protein intake, while the CC-genotype carriers presented significantly lower HbA1c (*p* = 0.033) and significantly higher CIR30 (*p* = 0.03). The linear regression models revealed that an increase in energy derived from proteins in TT carriers was associated with higher HbA1c levels (β = 0.37 (95% CI: 0.01–0.74, *p* = 0.05)), although, in general, carrying the TT genotype, but without considering protein intake, showed an opposite tendency—to lower HbA1c levels (β = −0.22 (95% CI: 0.47 to −0.01, *p* = 0.05). Among the subjects stratified to the lower quantile of carbohydrate intake, the TT-genotype individuals presented higher HbA1c (*p* = 0.041), and the CC-genotype subjects presented higher VAT (*p* = 0.033), lower SAT (*p* = 0.033), and higher VAT/SAT ratios (*p* = 0.034). In both the CC- and TT-genotype carriers, we noted higher VAT (*p* = 0.012 and *p* = 0.0006, respectively), lower SAT (*p* = 0.012 and *p* = 0.0006, respectively) and higher VAT/SAT ratios (*p* = 0.016 and *p* = 0.00062, respectively) when dietary fat provided more than 30% of total daily energy intake, without any differences in total body fat content. Our findings suggest that associations of the common TCF7L2 SNP with glucose homeostasis and obesity-related parameters may be dependent on daily macronutrient intake, which warrants further investigations in a larger population, as well as interventional studies.

## 1. Introduction

Type 2 diabetes mellitus (T2DM) has become an important medical problem [1]. T2DM patients are at higher risk of cardiovascular morbidity and mortality [2]; therefore, more effective prophylaxis and treatments are urgently required. Large-scale genome-wide association studies (GWAS) have revealed that one of the greatest genetic risk factors for T2DM is conferred by the TCF7L2 (transcription factor-7–like 2) gene [3,4]. The increased risk of T2DM conferred by genetic variations in TCF7L2 involves various mechanisms, including the modulation of the enteroinsular axis and enhanced expression of the gene in islets, affecting the function of β-cells and impairing insulin secretion [5,6,7]. Genetic variants of TCF7L2 confer a strong risk of T2DM by influencing the gene’s expression in pancreatic islets; it encodes for a transcription factor involved in Wnt signaling [8], which has shown to regulate pancreatic β-cell proliferation [9]. No significant differences in obesity-related parameters in relation to the TCF7L2 genetic variants have been found [3]; moreover, Corella D et al. found that the TCF7L2 genetic variants are more strongly associated with T2DM incidence in non-obese subjects [10]. The associations between T2DM risk and various single-nucleotide polymorphisms (SNPs) in the TCF7L2 gene were mainly found for rs7903146, rs7901695, and rs12255372, and these genetic variants are in strong linkage disequilibrium (LD) [11]. We also observed a very strong LD between TCF7L2 loci in our study population [12], so we focused on rs7901695 for further analysis. A meta-analysis conducted by Peng S et al. [11] confirmed a significant association of rs7901695 and T2DM risk (OR: 1.32; 95% CI: 1.25–1.39) and showed that the minor allele frequency (MAF) for rs7901695 ranges between 3.9 and 45.5% (mean: 21.9%), which may be different across various populations.

Although it is suggested that the TCF7L2 gene is involved in nearly a fifth of all T2DM cases [7], T2DM should not be considered only a genetic disorder, since most cases result from polygenic and multifactorial, especially gene–environmental interactions [4,13]. In our previous study, we noted lower glucose utilization [12] and an altered lipid metabolism [14] in healthy subjects carrying the high-risk CC genotype, rs7901695, of the TCF7L2 gene after consuming high-carbohydrate meals. Based on these observations, we hypothesized that dietary macronutrients may influence the risk of impaired glucose homeostasis development in carriers of the high-risk TCF7L2 genotype. The aim of our study was to evaluate whether daily macronutrient intake may alter the impact of the TCF7L2 gene on glucose homeostasis and obesity-related parameters.

## 2. Materials and Methods

The study is registered at ClinicalTrials.gov as NCT03792685, a meal-test intervention study, but the data presented in this manuscript were obtained from a cross-sectional study, at baseline, before subjects were included in the intervention sub-study.

### 2.1. Participants and Study Design

For this analysis, we included a population-based sample from the cohort study comprising Caucasian individuals (aged 18–79 y), whose characteristics were previously described in detail [15,16]. We analyzed results for 810 subjects (47.5% men and 52.5% women), who were 18–79 years old (mean age, 42.1 (±14.5) years). The mean BMI was 28.5 (±6.6) kg/m^2^ (min., 15.6 kg/m^2^; max., 56.5 kg/m^2^). Of the subjects, 33.9% had BMIs < 25.00 kg/m^2^; 34.5% were overweight, with BMIs ≥ 25.00 and <30.00 kg/m^2^; and 31.6% were obese, with BMIs ≥ 30.00 kg/m^2^. We included participants who did not have any endocrine, renal, hepatic or gastrointestinal disorders and did not report any treatments (including dietary supplements, following any specific eating pattern or diet, etc.) that might have affected the results (Supplementary Figure S1). Pregnant or breast-feeding women were excluded. The criteria were evaluated based on the available medical history, laboratory results, and exams performed during screening visits, as well as based on medical interviews with the participants.

### 2.2. Anthropometric and Body Composition Analysis

Body height was measured using the standard methods [17], by the same study team members. All the individuals underwent body weight and body composition measurements, including for total body fat content, fat-free mass (FFM), and skeletal muscle mass (SMM), assessed with the bioelectrical impedance method (InBody 220, Biospace, Seoul, Korea). The visceral adipose tissue (VAT) and subcutaneous adipose tissue (SAT) contents were measured by bioelectrical impedance analysis (Maltron 920-2 BioScan, Maltron International Ltd., Rayleigh, UK). The waist circumference was measured at a point midway between the lower rib and the iliac crest on the midaxillary line, and the hip circumference, at the level of the widest circumference over the great trochanters.

### 2.3. Blood Collection and Biochemical Analysis

All the participants were asked to fast for 8–12 h, and oral glucose tolerance tests (OGTTs) were performed. Venous blood samples were collected for measuring plasma glucose and insulin concentrations (0, 30, 60, and 120 min after glucose intake), and hemoglobin A1c (HbA1c) levels (0 min). The blood glucose and HbA1c concentrations were measured immediately after the specimens were drawn, while the blood samples for the insulin concentration measurements were centrifuged in accordance with the kit instructions, and the serum was stored at −20 °C until testing (approximately 3 months). To measure the insulin concentrations, we used an immunoradiometric assay (Insulin, IRMA, DiaSource, Ottignies-Louvain-la-Neuve, Belgium; Wallac Wizard 1470 Automatic Gamma Counter, PerkinElmer, Life Science, Turku, Finland). The plasma glucose concentrations were measured by the hexokinase enzymatic colorimetric assay (Cobas c111, Roche Diagnostics Ltd., Rotkreuz, Switzerland). The HbA1c was assessed using high-performance liquid chromatography (HPLC) (D-10 Hemoglobin Testing System, Bio-Rad Laboratories Inc. Hercules, CA, USA by France, Bio-Rad, Marnes-la-Coquette).

### 2.4. Calculations

The BMI was calculated as follows: body weight (kg)/height squared (m). The waist–hip ratio (WHR) was calculated by dividing the waist by hip circumference. The VAT/SAT ratio was calculated by dividing the visceral by subcutaneous adipose tissue. The area under the curve (AUC) was calculated using the trapezoid rule. Homeostatic model assessment of insulin resistance (HOMA-IR) was determined according to the following formula: (fasting plasma glucose concentration (mmol/L)) × (fasting insulin concentration (μU/mL))/22.5. The index homeostatic model assessment of β-cell function (HOMA-B) was calculated using the following formula: 20 × fasting insulin concentration (μIU/mL)/fasting glucose concentration (mmol/mL) −3.5. The corrected insulin response (CIR30) was calculated using the following formula: insulin 30′ concentration/(glucose 30′ concentration × (glucose 30′ concentration −70)). The metabolic equivalent (MET, minutes/week) was calculated using the following formula: (MET level) × (minutes of activity) × (events/ week).

### 2.5. Daily Physical Activity and Dietary Intake Analysis

Daily physical activity was evaluated with a self-administered questionnaire, IPAQ-LF (International Physical Activity Questionnaire-Long Form) [18]. The results were used to calculate MET values, based on which each participant was classified as having a low, moderate or high level of physical activity. The analyses of the 3-day food diaries were performed on the data from 662 subjects, because not all of the participants completed the diaries. The subjects were instructed to compare their portion sizes with the portion size and weight in color photographs, as well as to weigh food, if possible. The daily total intakes of energy, protein, carbohydrate, and fat were analyzed using the Dieta 6 software (National Food and Nutrition Institute, Warsaw, Poland).

In order to evaluate the interactions between genetic factors and dietary intake, the participants were divided into the two quantiles as follows: lower and upper quantiles of daily protein intake (≤18% and >18% of total energy intake, respectively), lower and upper quantiles of daily carbohydrate intake (≤48% and >48% of total energy intake, respectively), and lower and upper quantiles of daily fat intake (≤30% and >30% of total energy intake, respectively).

### 2.6. Genetic Analyses

We genotyped for the TCF7L2 SNP in rs7901695. The DNA was extracted from peripheral blood leukocytes, by a salting-out method described previously in detail [19]. Genotyping for the SNP was conducted using TaqMan SNP technology with a ready-to-use human assay library (Applied Biosystems, Waltham, MA, USA), using an OpenArray System (Life Technologies, Grand Island, NY, USA) as a tool for high-throughput genotyping. The genetic analyses were performed in duplicate in accordance with the manufacturer’s instructions. To detect possible false-positive signals caused by contamination, a negative control consisting of a sample without a template was used.

### 2.7. Statistical Analysis

Continuous data are summarized by the numbers of observations (N), arithmetic means, and standard deviations (SDs). For discrete data, we present the numbers of observations and frequencies (%). The subjects included in the study were divided into quantiles based on the average daily macronutrient (protein, carbohydrate and fat) intakes, with the thresholds set as the median value of each parameter. Continuous parameters were tested for normality with Shapiro–Wilk test and visual inspection. The homogeneity of the variance across groups was analyzed by Levene’s test. For response variables that failed the normality tests, we used nonparametric tests. The differences in selected parameters between subjects stratified by dietary macronutrient intake were evaluated using analysis of variance (ANOVA) or the Kruskal–Wallis test for the numerical variables, with either Tukey’s or Dunn’s post-hoc test with Holm *p*-value adjustment (in case multiple pairwise tests were performed or when there were multiple grouping variables, as presented in Table 1 and Figures), and chi-squared tests for categorical variables.

To test the hypothesis that the associations between the TCF7L2 rs7901695 genetic variants and continuous responses varied in groups according to stratification by daily protein, fat, and carbohydrate intake, we added (dietary macronutrient quantile) × (TCF7L2 rs7901695 genotype) interaction terms to the multivariate linear regression models. We observed that the association between the investigated genotypes and variables describing body composition (weight, BMI and fat-free mass) and the subject’s glycemic status varied in different dietary groups, confirming the hypothesis that the effects of dietary factors and the genotype interact. Using boxplots in the figures, we present the differences in the median values of the selected responses and the interquartile ranges (IQRs) in different genetic and dietary strata. These results were statistically significant after adjustment for the age, sex, BMI (if applicable), total daily energy intake, and physical activity level. The statistical significance level was set at <0.05 for all two-sided tests and multivariate comparisons. All of the calculations and analyses were performed in R (version 4.0.2) [20].

## 3. Results

Based on criteria for diabetes diagnosis [21], 411 participants (50.2%) had prediabetes or diabetes, 109 participants (13.3%) reported previously known histories of prediabetes or diabetes, and 56 individuals (6.9%) were being treated with anti-diabetic medications. Individuals who received anti-diabetic or lipid-lowering medications (47 individuals, 5.8% of the participants) were not included in this analysis, as these were potential confounders. The clinical characteristics of the studied population, stratified by the investigated TCF7L2 rs7901695 genotypes, are presented in Table 1. No significant deviation from the Hardy–Weinberg equilibrium was observed (Table 1). The frequencies of overweight/obesity and prediabetes/diabetes prevalence did not differ according to genotype (Table 1). We did not find any significant differences in BMI, total body fat content, WHR, body fat distribution, blood glucose and insulin concentrations, glucose and insulin AUCs during OGTTs, and HbA1c levels (Table 1). We did not observe any differences between the investigated genotypes in the daily total energy and macronutrient intakes or in the daily physical activity levels (Table 1).

### 3.1. Dietary Assessment

We did not notice any differences in daily energy and macronutrient intake between the studied genotypes (Table 1).

### 3.2. Associations between the rs7901695 Polymorphism, Glucose Homeostasis, Obesity-Related Parameters, and Dietary Protein Intake

**Figure 1:**
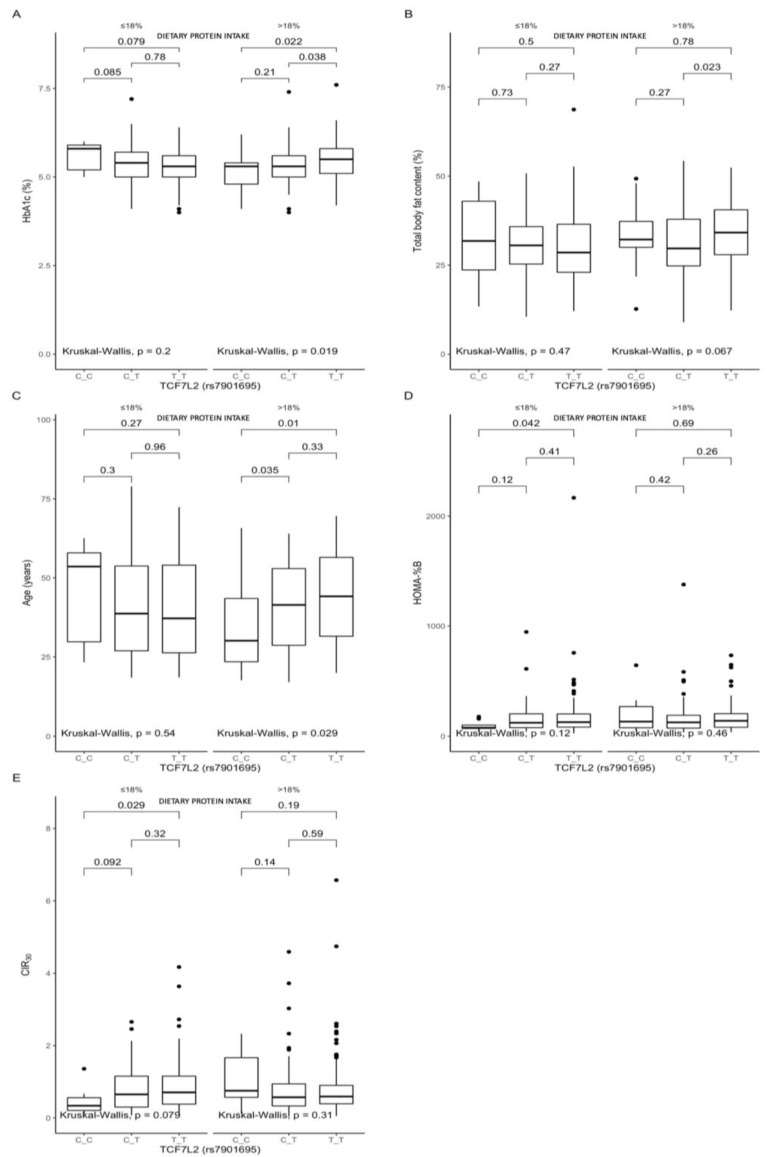
Association of the TCF7L2 rs7901695 genotypes with (**A**) HbA1c (%), (**B**) total body fat content (%), (**C**) age (years), (**D**) HOMA-B, and (**E**) CIR30 by dietary protein intake strata: ≤18% and >18% of total daily energy intake. The differences in median values of the selected responses and the interquartile ranges (IQRs) in different genotypic and dietary strata are presented. Kruskal–Wallis test *p*-values for the differences in median values within three strata are presented.

A comparison between the carriers of the different genotypes, stratified by macronutrient intake, showed that, among the participants in the upper protein intake quantiles, the TT-genotype carriers presented significantly higher HbA1c (Figure 1A) than the CT- and CC-genotype participants (*p* = 0.038 and *p* = 0.022, respectively). We also noted higher total body fat contents (Figure 1B), but the difference was observed only between the TT- and CT-genotype subjects (*p* = 0.023). Among the subjects from the upper protein intake quantiles, the CC-genotype subjects were significantly younger (Figure 1C) than the CT- and TT-genotype carriers (*p* = 0.035 and *p* = 0.01, respectively). The linear regression models that included the covariates age, sex, and total energy intake, as well as the (dietary protein quantile) × (TCF7L2 genotype) interaction term, showed a marginally significant interaction effect of TCF7L2 genotype and macronutrients on HbA1c levels. The increase in energy derived from proteins in TT carriers was associated with higher HbA1c levels (β = 0.37 (95% CI: 0.01–0.74, *p* = 0.05)), although, in general, carrying the TT genotype (without considering protein intake) showed an opposite tendency to lower HbA1c levels (β = −0.22 (95% CI: 0.47 to −0.01, *p* = 0.05). Moreover, the CC-genotype carriers stratified to the lower protein intake quantiles presented significantly lower HOMA-B (*p* = 0.042, Figure 1D) and CIR30 (*p* = 0.029, Figure 1E).

**Figure 2:**
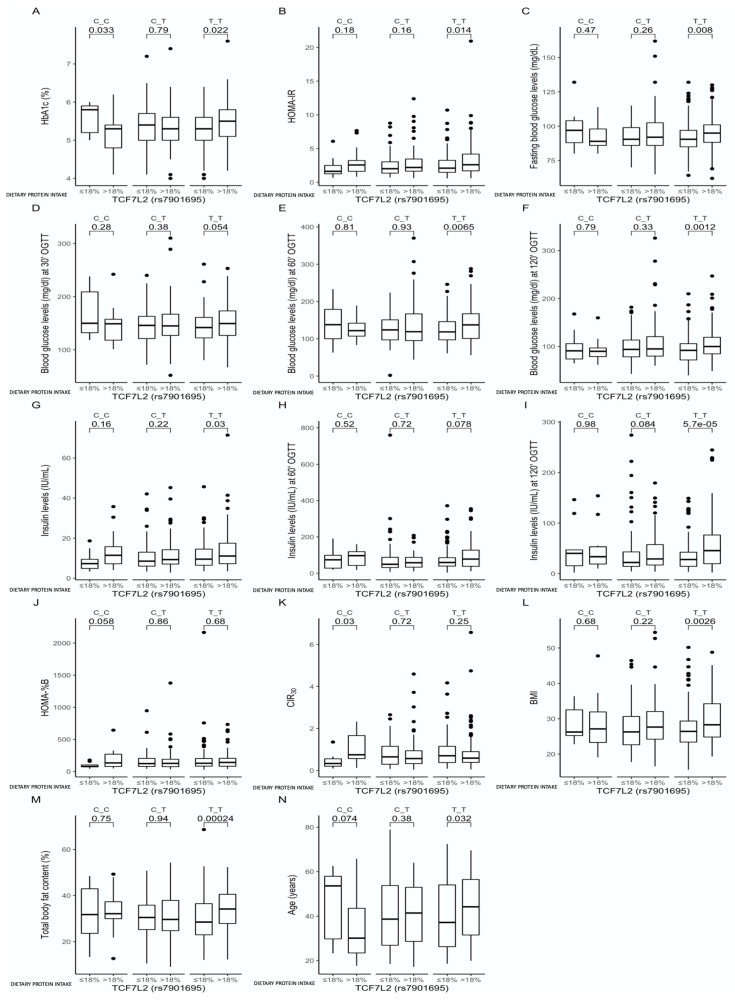
Association of dietary protein intake ≤18% and > 18% of total daily energy intake with (**A**) HbA1c (%), (**B**) HOMA-IR, (**C**) fasting blood glucose levels (mg/dL), (**D**) blood glucose levels at 30 min of OGTT (mg/dL), (**E**) blood glucose levels at 60 min of OGTT (mg/dL), (**F**) blood glucose levels at 120 min of OGTT (mg/dL), (**G**) fasting insulin levels (IU/mL), (**H**) insulin levels at 60 min of OGTT (IU/mL), (**I**) insulin levels at 120 min of OGTT (IU/mL), (**J**) HOMA-B, (**K**) CIR30, (**L**) BMI (kg/m^2^), (**M**) total body fat content (%), and (**N**) age (years), in the *TCF7L2* rs7901695 genotype carriers. The differences in median values of the selected responses and the interquartile ranges (IQRs) in different genotypic and dietary strata are presented.

The analysis among carriers of the same genotype stratified according to daily protein intake showed that the CC-genotype participants who were stratified to the lower protein intake quantiles presented higher HbA1c levels (*p* = 0.033, Figure 2A), while the TT-genotype carriers showed the inverse: those stratified to the higher protein intake quantiles had higher HbA1c levels (*p* = 0.022, Figure 2A). The TT-genotype carriers stratified to the upper protein intake quantiles also presented higher HOMA-IR (*p* = 0.014, Figure 2B), higher blood glucose concentrations at every time point of the OGTTs (*p* = 0.008, *p* = 0.054, *p* = 0.0065 and *p* = 0.012, respectively; Figure 2C–F), and higher insulin concentrations at fasting and during OGTTs (*p* = 0.03 and *p* = 5.7 × 10^−5^; Figure 2G–I). The CC-genotype carriers stratified to the upper protein intake quantiles presented significantly lower HbA1c (*p* = 0.033, Figure 2A), marginally significantly higher HOMA-B (*p* = 0.058, Figure 2J), and significantly higher CIR30 (*p* = 0.03, Figure 2K). Moreover, in the TT-genotype carriers, we noted that those stratified to the upper protein intake quantiles showed higher BMIs (*p* = 0.0026, Figure 2L) and total body fat contents (*p* = 0.00024, Figure 2M) and were significantly older than the TT-genotype carriers from the lower protein intake quantiles (*p* = 0.032, Figure 2N).

### 3.3. Associations between the rs7901695 Polymorphism, Glucose Homeostasis, Obesity-Related Parameters, and Dietary Carbohydrate Intake

**Figure 3:**
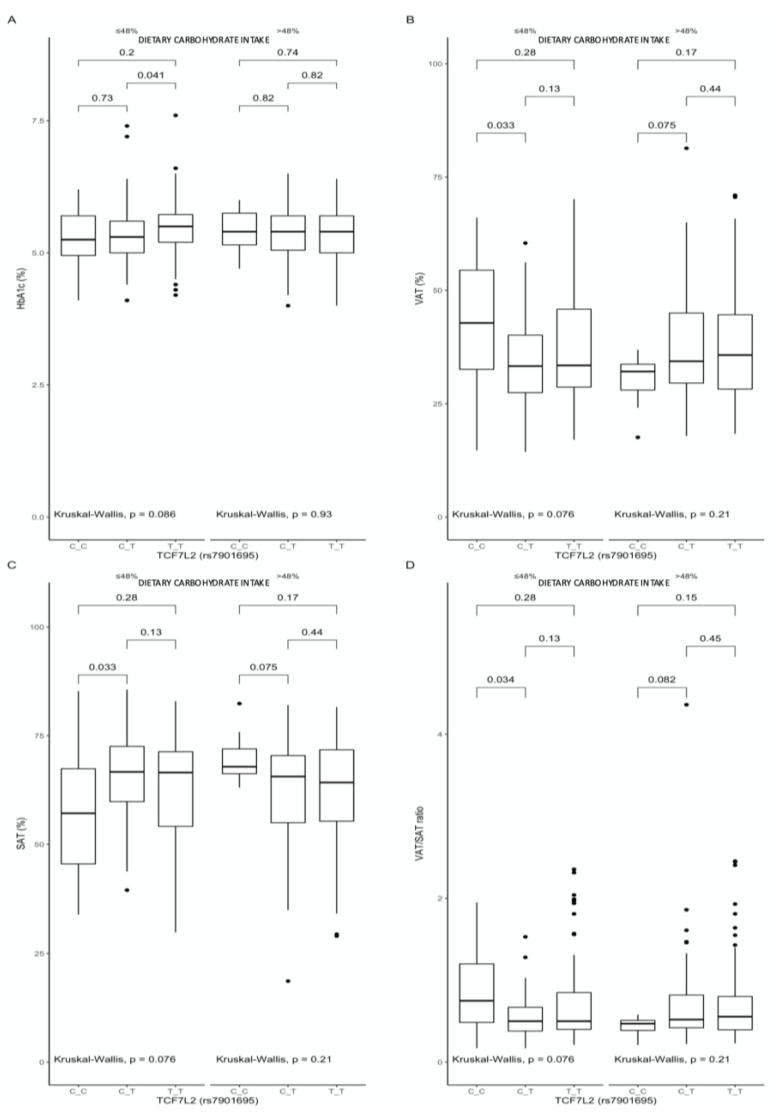
Association of the TCF7L2 rs7901695 genotypes with (**A**) HbA1c (%), (**B**) VAT (%), (**C**) SAT (%), and (**D**) VAT/SAT ratio, by dietary carbohydrate intake strata: ≤48% and >48% of total daily energy intake. The differences in median values of the selected responses and the interquartile ranges (IQRs) in different genotypic and dietary strata are presented. Kruskal–Wallis test *p*-values for the differences in median values within three strata are presented.

A comparison between the carriers of the different genotypes, stratified by macronutrient intake, showed that, among the subjects stratified to the lower quantile of carbohydrate intake, the TT-genotype individuals presented higher HbA1c (*p* = 0.041, Figure 3A), while the CC-genotype subjects presented higher VAT (*p* = 0.033, Figure 3B), lower SAT (*p* = 0.033, Figure 3C), and higher VAT/SAT ratios (*p* = 0.034, Figure 3D).

**Figure 4:**
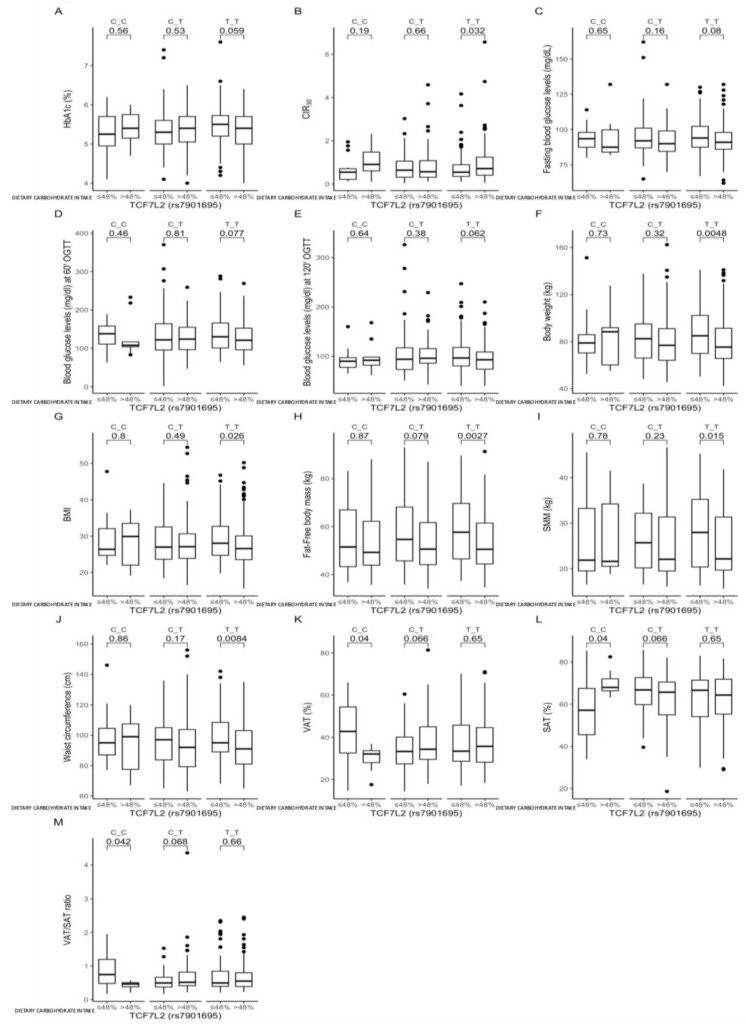
Association of dietary carbohydrate intakes ≤48% and >48% of total daily energy intake with (**A**) HbA1c, (**B**) CIR30, (**C**) fasting blood glucose levels (mg/dL), (**D**) blood glucose levels at 60 min of OGTT (mg/dL), (**E**) blood glucose levels at 120 min of OGTT (mg/dL), (**F**) body weight (kg), (**G**) BMI (kg/m^2^), (**H**) FFM (kg), (**I**) SMM (kg), (**J**) waist circumference (cm), (**K**) VAT (%), (**L**) SAT (%), and (**M**) VAT/SAT ratio, in the TCF7L2 rs7901695 genotype carriers. The differences in median values of the selected responses and the interquartile ranges (IQRs) in different genotypic and dietary strata are presented.

Further comparisons between carriers of the same genotype showed a tendency for higher HbA1c levels (*p* = 0.059, Figure 4A) and significantly lower CIR30 values (*p* = 0.032, Figure 4B) among those TT-genotype carriers who were stratified to the lower quantiles of carbohydrate intake. These subjects also presented significantly higher body weights (*p* = 0.0048, Figure 4F), BMIs (*p* = 0.026, Figure 4G), FFM (*p* = 0.0027, Figure 4H), SMM (*p* = 0.015, Figure 4I), and waist circumferences (*p* = 0.0084, Figure 4J). We noted that, when the daily energy intake derived from carbohydrates >48%, then the CC-genotype subjects presented significantly higher VAT (*p* = 0.04, Figure 4K), lower SAT (*p* = 0.04, Figure 4L), and higher VAT/SAT ratios (*p* = 0.042, Figure 4M).

### 3.4. Associations between the rs7901695 Polymorphism, Glucose Homeostasis, Obesity-Related Parameters, and Dietary Fat Intake

**Figure 5:**
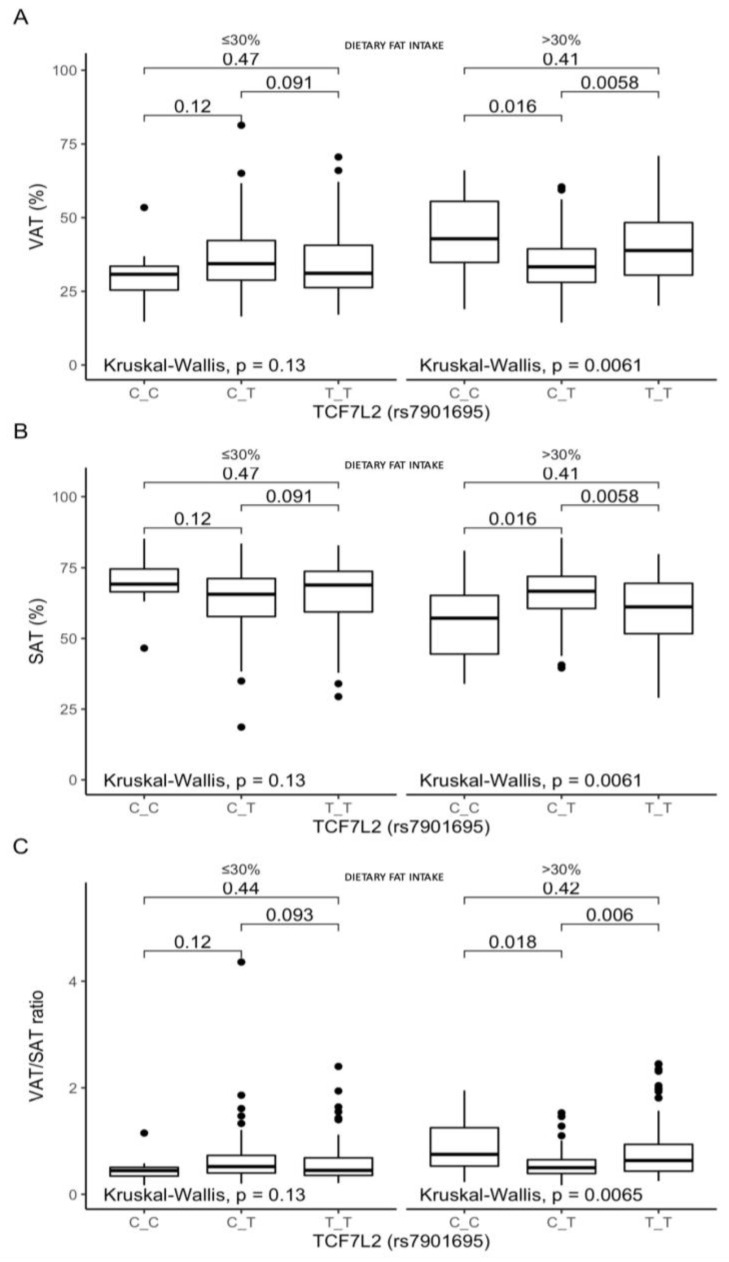
Association of the TCF7L2 rs7901695 genotypes with (**A**) VAT (%), (**B**) SAT (%), and (**C**) VAT/SAT ratio, by dietary fat intake strata: ≤30% and >30% of total daily energy intake. The differences in median values of the selected responses and the interquartile ranges (IQRs) in different genotypic and dietary strata are presented. Kruskal–Wallis test *p*-values for the differences of median values within three strata are presented.

The stratification of the subjects according to dietary fat intake revealed differences in the body fat distribution between the heterozygous CT-genotype carriers and homozygous carriers of both genotypes. Among the participants stratified to the upper fat intake quantiles, the CT-genotype individuals presented lower VAT than the CC- and TT-genotype carriers (*p* = 0.016 and *p* = 0.0058, respectively; Figure 5A), higher SAT (*p* = 0.016 and *p* = 0.0058, respectively; Figure 5B), and lower VAT/SAT ratios (*p* = 0.018 and *p* = 0.006, respectively; Figure 5C).

**Figure 6:**
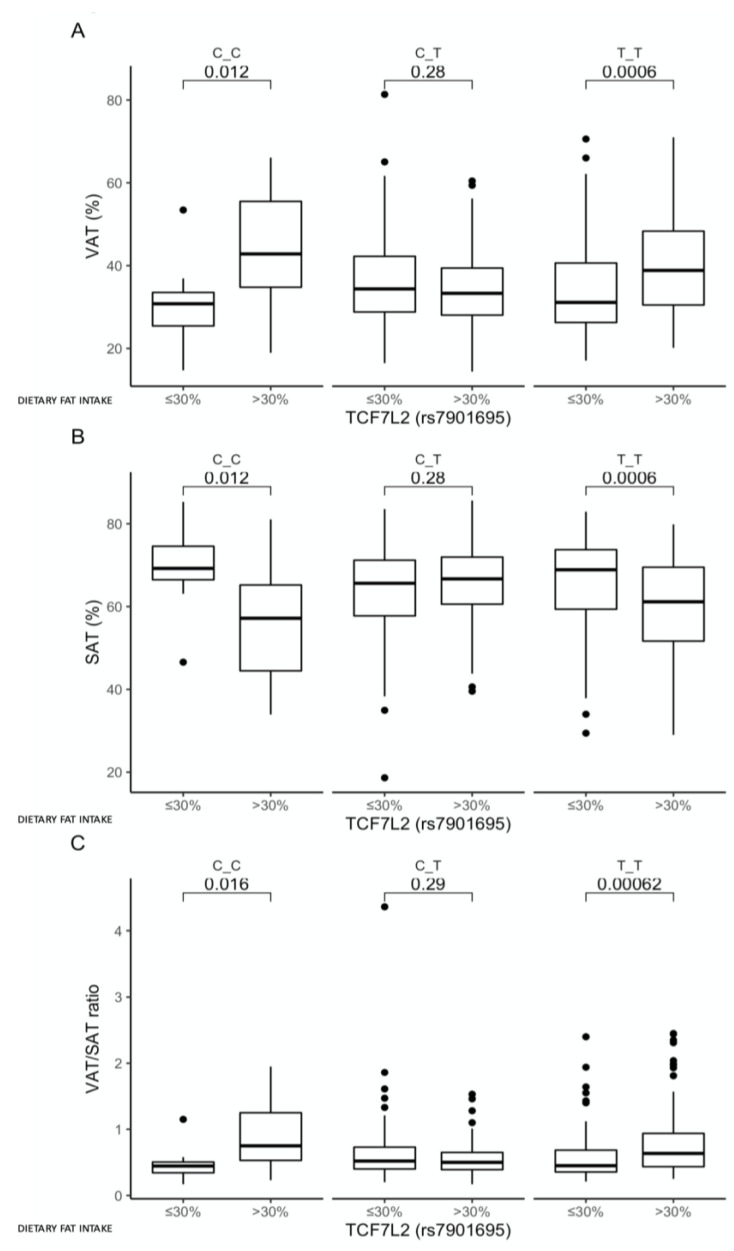
Association of dietary fat intake ≤30% and >30% of total daily energy intake with (**A**) VAT (%), (**B**) SAT (%), and (**C**) VAT/SAT ratio, in the TCF7L2 rs7901695 genotype carriers. The differences in median values of the selected responses and the interquartile ranges (IQRs) in different genotypic and dietary strata are presented.

Comparison between carriers of the same genotype showed that, for both the CC- and TT-genotype carriers, higher VAT (*p* = 0.012 and *p* = 0.0006, respectively; Figure 6A), lower SAT (*p* = 0.012 and *p* = 0.0006, respectively; Figure 6B), and higher VAT/SAT ratios (*p* = 0.016 and *p* = 0.00062, respectively; Figure 6C) were presented when dietary fat provided >30% of the total daily energy intake. We did not observe any other associations between dietary fat intake, genotypes, and the other investigated parameters.

## 4. Discussion

Our study indicates that the effects of TCF7L2 rs7901695 genetic variants on glucose homeostasis and obesity-related parameters may depend on daily macronutrient intake. We did not observe any significant differences according to genotype in the clinical characteristics of the studied group. In non-Amish Caucasian subjects, during intravenous glucose tolerance tests (IVGTTs), the associations between rs7901695 variants and insulin sensitivity (Si), as well as the disposition index (Di), were found by Damcott CM et al. [22]. The lower Si and lower Di suggest both a reduction in insulin sensitivity and a failure of the β-cells to fully compensate for the insulin resistance. The discrepancies between their results and ours could be due to the different methods used—Damcott CM et al. performed IVGTTs, while we performed OGTTs—and we should consider the role of incretin’s effects, especially because it has also been shown to be associated with TCF7L2 genetic variants [23]. The fact that we did not observe any crucial differences according to genotype until we included dietary factors in the analysis may be considered one of the reasons for the conflicting findings from different studies regarding the association of the TCF7L2 genetic variants with obesity and glucose homeostasis [24,25,26], and may suggest that dietary factors could be involved in these associations.

We noted significantly higher HbA1c in the TT subjects when >18% of the daily energy intake was derived from proteins, which might have been a result of higher total body fat content, but it was only significant when compared to the CT genotype, while between the CC- and TT-genotype participants, we did not observe any differences in total body fat content. The CC-genotype carriers were significantly younger, which could also have affected our results. Analysis among carriers of the same genotype stratified according to dietary protein intake showed that the CC-genotype carriers in the lower protein intake quantiles presented higher HbA1c levels, while the TT-genotype carriers presented higher HOMA-IR, blood glucose and insulin concentrations when dietary protein provided >18% of the total energy intake. These observations might have resulted from the noted higher BMIs and total body fat contents in these subjects, as well as from the fact that these individuals were significantly older [27]. Therefore, we decided to construct linear regression models, including adjustment for the possible covariates, and we noted a marginally significant interaction. The increase in energy derived from proteins by the TT-genotype carriers was associated with higher HbA1c levels, which is the most intriguing finding, because the carriage of the TT genotype without considering the macronutrient intake showed an opposite tendency. Moreover, if dietary proteins provided ≤18% of daily energy intake, the CC-genotype carriers presented significantly lower CIR30 levels, indicating lower β-cell activity [28,29]. We did not notice any differences in ß-cell activity between the CC, CT, and TT genotypes when dietary proteins provided >18% of daily energy intake, which is also very intriguing. The CC-genotype carriers stratified to the upper protein intake quantiles presented significantly lower HbA1c and significantly higher CIR30 than those in the other quantiles. It is important to mention that the CC-genotype carriers from the upper protein intake quantiles showed a tendency to be younger, but the linear regression models, after adjustment for age, confirmed that the increase in energy derived from proteins for the CC carriers showed a tendency to be associated with lower HbA1c levels; while carrying the CC genotype, without considering the daily protein intake, showed an opposite tendency. We did not find any studies that had investigated these associations, but it has been reported that the other TCF7L2 loci (the HapA (rs7903146 C) allele and the rs10885406 A allele) attenuate the positive association between animal protein intake and body weight gain and that some carriers are apparently less sensitive to an increase in dietary protein intake [30].

We notably found that carbohydrates may have an impact mostly on the obesity-related parameters, dependently on the carried genotype of TCF7L2 rs7901695. The TT-genotype carriers presented significantly higher HbA1c levels, higher VAT, lower SAT, and higher VAT/SAT ratios than the heterozygous CT-genotype subjects, when dietary carbohydrates provided ≤48% of daily energy intake. The distribution of body fat tissue is of crucial importance, because the visceral adipocytes are more active and their metabolic activity may lead to the development of insulin resistance, metabolic disturbances, and increased all-cause mortality [31,32]. A higher VAT/SAT ratio may be associated with increased metabolic and cardiovascular risk, independently of BMI and visceral fat content [33].

The comparisons between carriers of the same genotypes stratified according to carbohydrate intake showed that the TT-genotype subjects presented marginally significantly lower HbA1c and significantly higher CIR30 when carbohydrates provided >48% of total daily energy intake. This might result from significantly lower body weight, but these parameters were associated with lower FFM and SMM, without any differences in total body fat content and distribution. Lower muscle mass is also associated with lower insulin sensitivity and disturbances in glucose homeostasis [15,34]. Nevertheless, the TT-genotype carriers, despite having lower fat-free mass and muscle mass, presented more favorable results for glucose homeostasis parameters when dietary carbohydrates provided >48% of daily energy intake. On the other hand, we noted that CC-genotype carriers who derived ≤48% of their daily energy from carbohydrates presented higher VAT, lower SAT, and higher VAT/SAT ratios, suggesting that the restriction of dietary carbohydrates should not be recommended for carriers of this genotype. Based on our previous results from the intervention studies [12,14], we expected to find significant associations between dietary carbohydrate intake for high-risk genotypes, which could be crucial for T2DM development; however, the meals that we used in our experiments differed in dietary protein and fat contents. The adverse metabolic results we observed after the high-carbohydrate—which was, at the same time, the low-protein—meal could explain our observations. Taken together, our results indicate that, in carriers of the protective TT genotype of TCF7L2 rs7901695, following a diet with >48% of daily energy derived from carbohydrates may enhance the protective effects of the genotype, while in individuals with the high-risk CC genotype, carbohydrate intake may not have any crucial impact on glucose homeostasis. It has already been shown that some dietary behaviors may only be associated with protection from T2DM among non-risk-allele carriers [35]. We did not find any other results with which to compare our observations; however, Cornelis MC et al. [36], who investigated the other TCF7L2 SNP, reported that the risk of T2DM associated with rs12255372 did not significantly differ by carbohydrate intake, but it may be modified by the carbohydrate quality and quantity, which was not the aim of our study, and further analyses are needed.

We did not find any crucial associations with dietary fat intake, except that a daily fat intake providing >30% of total energy in the homozygous CC and TT carriers was unfavorably associated with higher VAT, lower SAT, and higher VAT/SAT ratios. We did not find any studies investigating these associations with the TCF7L2 rs7901695 SNP, but studies on the other genetic loci show that individuals carrying the TCF7L2 rs12255372 risk genotype may be able to reduce their body adiposity by following a low-fat diet [37], and subjects who are homozygous for the TCF7L2 rs7903146 T-risk allele are more sensitive to hypoenergetic diets with low fat contents [38]. Moreover, it was shown that the risk of metabolic syndrome associated with TCF7L2 rs7903146 is augmented by dietary saturated fatty acid intake, with a further impairment of insulin sensitivity [39], which indicates that further analyses are required.

To the best of our knowledge, this is the first study investigating the associations between rs7901695 genetic variants and macronutrient intake, as well as the effect of these relationships on glucose homeostasis and obesity-related parameters. The major strength of our study is that it was based on a relatively large population-based sample and that the participants included in the study group did not differ in daily total energy and macronutrient intakes, or in daily physical activity levels; therefore, we can exclude the potential impacts of different macronutrient intakes and physical activity on gene expression and the activation of pathways that could have influenced our results. Nevertheless, we must be very cautious when extrapolating our results because our study does have some potential limitations, a major one being the relatively low number of risk genetic variant carriers, due to the low genotype frequency in the general population. Therefore, further studies in larger and more diverse populations are needed to verify our findings. This might explain the lack of differences according to genotype in the ratios of prediabetes or diabetes in the studied population. Another key limitation is collider bias, which could have been prevented by applying appropriate inclusion criteria. The next limitation is the fact that the dietary data were based on self-reported diaries, and it has been demonstrated that people may underreport their food intake [40]. However, the diaries and dietary questionnaires are the only tools that are available for estimating daily dietary intake in large population studies. Another limitation is the fact that we did not use accelerometers for the physical activity evaluation; nevertheless, the long version of IPAQ is a validated and acceptable method with which to verify daily physical activity levels.

## 5. Conclusions

Our study suggests that, in carriers of the high-risk TCF7L2 genotype, focusing only on dietary carbohydrates and fats may be insufficient to prevent T2DM and that an important role may also be played by dietary protein intake. These observations are very intriguing, especially given the current interest in carbohydrate-restricted diets among the general population. Moreover, our study showed that carbohydrate restriction may have an adverse impact on subjects carrying the protective genotype variants, as could increasing dietary protein intake. The practical implications of our results might be to recommend diets with >18% of daily energy derived from dietary protein for CC-genotype carriers in order to limit visceral adipose tissue deposition and that carbohydrates should provide >48% of total daily energy intake.

## Figures and Tables

**Table 1:** Characteristics of participants stratified by rs7901695 genotype.

rs7901695	T/T	C/T	C/C	*p*-Value
N	442	317	51	
Men/women (%)	48/52	49/51	43/57	
Genotype frequency	54.57%	39.14%	6.30%	>0.05
Age (years)	40.94	40.93	40.85	0.942
BMI (kg/m^2^)	28.7 (6.6)	28.2 (6.8)	28.3 (6.4)	0.574
BMI < 25.0 (kg/m^2^)	143 (32.6%)	111 (35.9%)	16 (32.0%)	0.856
BMI 25.0–29.9 (kg/m^2^)	153 (34.9%)	105 (34.0%)	16 (32.0%)	
BMI ≥ 30.0 (kg/m^2^)	142 (32.4%)	93 (30.1%)	18 (36.0%)	
Total body fat content (%)	31.7 (9.8)	30.8 (9.3)	32.7 (9.9)	0.272
WHR	0.932 (0.086)	0.925 (0.091)	0.915 (0.087)	0.325
Visceral fat content (%)	37.8 (12.8)	36.3 (10.8)	36.7 (13.1)	0.732
Subcutaneous fat content (%)	62.1 (13.2)	63.7 (10.8)	63.3 (13.1)	0.738
Visceral/subcutaneous fat ratio	0.698 (0.465)	0.633 (0.419	0.661 (0.419)	0.732
Frequency of prediabetes or diabetes				
Yes	223 (50.5%)	159 (50.2%)	26 (51.0%)	0.992
No	219 (49.5%)	158 (49.8%)	25 (49.0%)	
Fasting blood glucose (mg/dl)	96.4 (26.4)	96.1 (19.9)	104.5 (31.8)	0.613
Blood glucose at 30′ of OGTT (mg/dl)	146.9 (34.5)	145.4 (36.2)	154.3 (51.7)	0.811
Blood glucose at 60′ of OGTT (mg/dl)	131.5 (47.7)	130.6 (48.8)	137.1 (63.4)	0.913
Blood glucose at 120′ of OGTT (mg/dl)	99.3 (33.0)	99.5 (36.0)	101.0 (58.3)	0.660
Glucose AUC during OGTT	0.22 (95% CI: 0.21–0.23)	0.22 (95% CI: 0.21–0.23)	0.23 (95% CI: 0.19–0.28)	0.349
HbA1c	5.6 (1.1)	5.5 (1.1)	5.6 (1.1)	0.427
Fasting insulin (IU/mL)	12.8 (9.3)	12.1 (10.4)	11.1 (7.0)	0.072
Insulin AUC during OGTT	0.14 (95% CI: 0.13–0.15)	0.13 (95% CI: 0.12–0.14)	0.4 (95% CI: 0.10–0.17)	0.118
HOMA-IR	3.1 (2.6)	2.9 (2.7)	2.8 (2.2)	0.084
HOMA-B	162.5 (218.9)	157.6 (152.8)	142.7 (106.4)	0.287
CIR_30_	0.9 (0.9)	0.8 (0.7)	0.8 (0.8)	0.495
Daily energy intake (kcal)	1814.7 (702.7)	1779.3 (708.6)	1610.1 (538.1)	0.216
% of daily energy from carbohydrates	48.0 (8.7)	47.0 (9.0)	47.2 (5.4)	0.329
% of daily energy from protein	18.8 (4.6)	19.1 (5.2)	19.9 (3.6)	0.334
% of daily energy from fat	31.1 (7.5)	31.4 (7.7)	31.2 (5.2)	0.154
Protein intake (n in the low/high quantiles)	140/128	92/94	10/20	
Carbohydrate intake (n in the low/high quantiles)	123/145	100/86	19/11	
Fat intake (n in the low/high quantiles)	133/135	95/91	14/16	
Daily physical activity level, n (%)				
Low	28 (6.3%)	28 (8.8%)	3 (5.9%)	0.246
Moderate	89 (20.1%)	65 (20.5%)	16 (31.4%)	
High	325 (73.5%)	224 (70.7%)	32 (62.7%)	

Data represent arithmetic mean values and standard deviations (SDs).

## Data Availability

The datasets used and/or analyzed during the current study are available from the corresponding author on reasonable request.

## Supplementary Materials

The following are available online at https://www.mdpi.com/article/10.3390/nu13061936/s1, Figure S1: Study flow chart diagram. Table S1: Power analysis for TCF7L2 (rs7901695) genotypes.
