# Supplementary material for: Dietary Macronutrient Intake May Influence the Effects of TCF7L2 rs7901695 Genetic Variants on Glucose Homeostasis and Obesity-Related Parameters: A Cross-Sectional Population-Based Study
Source: Nutrients. 2021 Jun 4;13(6):1936. doi: 10.3390/nu13061936 (PMC8230266; doi:10.3390/nu13061936)
Supplement: Supplementary file 1 [file nutrients-13-01936-s001.zip › Supplementary Table S1.pdf]

Supplementary Table S1. Power analysis for TCF7L2 (rs7901695) genotypes.

Parameter	Group 1	Group 2	N Group 1	N Group 2	Effect Size	Sig. Level	Power	Alternative
Age	C_C	C_T	60	374	0,1611	0,05	0,2115	two.sided
Age	C_C	T_T	60	547	0,1643	0,05	0,2263	two.sided
Age	C_T	T_T	374	547	0,0062	0,05	0,0510	two.sided
BMI	C_C	C_T	50	309	0,0199	0,05	0,0519	two.sided
BMI	C_C	T_T	50	438	0,0501	0,05	0,0629	two.sided
BMI	C_T	T_T	309	438	0,0695	0,05	0,1543	two.sided
Total body fat content	C_C	C_T	50	303	0,1987	0,05	0,2547	two.sided
Total body fat content	C_C	T_T	50	437	0,1022	0,05	0,1049	two.sided
Total body fat content	C_T	T_T	303	437	0,0908	0,05	0,2283	two.sided
WHR	C_C	C_T	50	307	0,1042	0,05	0,1047	two.sided
WHR	C_C	T_T	50	437	0,1910	0,05	0,2479	two.sided
WHR	C_T	T_T	307	437	0,0795	0,05	0,1870	two.sided
Visceral fat content	C_C	C_T	37	193	0,0365	0,05	0,0547	two.sided
Visceral fat content	C_C	T_T	37	275	0,0898	0,05	0,0805	two.sided
Visceral fat content	C_T	T_T	193	275	0,1299	0,05	0,2816	two.sided
Subcutaneous fat content	C_C	C_T	37	193	0,0361	0,05	0,0546	two.sided
Subcutaneous fat content	C_C	T_T	37	275	0,0873	0,05	0,0787	two.sided
Subcutaneous fat content	C_T	T_T	193	275	0,1267	0,05	0,2704	two.sided
VAT/SAT	C_C	C_T	37	193	0,0662	0,05	0,0656	two.sided
VAT/SAT	C_C	T_T	37	275	0,0823	0,05	0,0755	two.sided
VAT/SAT	C_T	T_T	193	275	0,1469	0,05	0,3451	two.sided
Fasting blood glucose	C_C	C_T	44	275	0,1727	0,05	0,1855	two.sided
Fasting blood glucose	C_C	T_T	44	391	0,1707	0,05	0,1883	two.sided
Fasting blood glucose	C_T	T_T	275	391	0,0177	0,05	0,0558	two.sided
Blood glucose at 30′	C_C	C_T	40	267	0,2370	0,05	0,2859	two.sided
Blood glucose at 30′	C_C	T_T	40	375	0,2022	0,05	0,2282	two.sided
Blood glucose at 30′	C_T	T_T	267	375	0,0499	0,05	0,0954	two.sided
Blood glucose at 60′	C_C	C_T	40	266	0,1443	0,05	0,1356	two.sided
Blood glucose at 60′	C_C	T_T	40	376	0,1189	0,05	0,1100	two.sided
Blood glucose at 60′	C_T	T_T	266	376	0,0294	0,05	0,0655	two.sided
Blood glucose at 120′	C_C	C_T	42	271	0,0609	0,05	0,0655	two.sided

Blood glucose at 120′	C_C	T_T	42	383	0,0598	0,05	0,0656	two.sided
Blood glucose at 120′	C_T	T_T	271	383	0,0072	0,05	0,0509	two.sided
HbA1c	C_C	C_T	44	273	0,2555	0,05	0,3478	two.sided
HbA1c	C_C	T_T	44	391	0,2146	0,05	0,2704	two.sided
HbA1c	C_T	T_T	273	391	0,0581	0,05	0,1138	two.sided
Fasting insulin	C_C	C_T	44	275	0,1016	0,05	0,0956	two.sided
Fasting insulin	C_C	T_T	44	392	0,1817	0,05	0,2071	two.sided
Fasting insulin	C_T	T_T	275	392	0,0588	0,05	0,1159	two.sided
CIR30	C_C	C_T	39	253	0,0396	0,05	0,0561	two.sided
CIR30	C_C	T_T	39	364	0,0633	0,05	0,0662	two.sided
CIR30	C_T	T_T	253	364	0,1025	0,05	0,2397	two.sided
HOMA-B	C_C	C_T	44	274	0,1250	0,05	0,1196	two.sided
HOMA-B	C_C	T_T	44	390	0,1869	0,05	0,2165	two.sided
HOMA-B	C_T	T_T	274	390	0,0710	0,05	0,1466	two.sided
HOMA-IR	C_C	C_T	44	274	0,0436	0,05	0,0583	two.sided
HOMA-IR	C_C	T_T	44	391	0,1073	0,05	0,1034	two.sided
HOMA-IR	C_T	T_T	274	391	0,0541	0,05	0,1053	two.sided
Type 2 diabetes	C_C	C_T	49	305	0,0035	0,05	0,0501	two.sided
Type 2 diabetes	C_C	T_T	49	433	0,0444	0,05	0,0599	two.sided
Type 2 diabetes	C_T	T_T	305	433	0,0402	0,05	0,0836	two.sided
Prediabetes-Impaired fasting glucose	C_C	C_T	49	303	0,0722	0,05	0,0754	two.sided
Prediabetes-Impaired fasting glucose	C_C	T_T	49	426	0,0775	0,05	0,0806	two.sided
Prediabetes-Impaired fasting glucose	C_T	T_T	303	426	0,1500	0,05	0,5134	two.sided
Prediabetes-Impaired glucose tolerance	C_C	C_T	49	303	0,2277	0,05	0,3140	two.sided
Prediabetes-Impaired glucose tolerance	C_C	T_T	49	426	0,1983	0,05	0,2590	two.sided
Prediabetes-Impaired glucose tolerance	C_T	T_T	303	426	0,0218	0,05	0,0596	two.sided
Daily Energy intake	C_C	C_T	30	186	0,2458	0,05	0,2376	two.sided
Daily Energy intake	C_C	T_T	30	268	0,2972	0,05	0,3370	two.sided
Daily Energy intake	C_T	T_T	186	268	0,0502	0,05	0,0822	two.sided
Protein intake	C_C	C_T	30	186	0,1589	0,05	0,1267	two.sided
Protein intake	C_C	T_T	30	268	0,2315	0,05	0,2240	two.sided
Protein intake	C_T	T_T	186	268	0,0506	0,05	0,0826	two.sided
Fat intake	C_C	C_T	30	186	0,0218	0,05	0,0514	two.sided
Fat intake	C_C	T_T	30	268	0,0126	0,05	0,0505	two.sided

Fat intake	C_T	T_T	186	268	0,0334	0,05	0,0641	two.sided
Carbohydrate intake	C_C	C_T	30	186	0,0241	0,05	0,0517	two.sided
Carbohydrate intake	C_C	T_T	30	268	0,0967	0,05	0,0792	two.sided
Carbohydrate intake	C_T	T_T	186	268	0,1160	0,05	0,2284	two.sided

N Group 1 and N Group 2 are the sample sizes. For all the two-sided tests, the effect size was calculated as the differences of Group 1 and Group 2 means divided by the common error variance. A significance level of 0.05 was set for all the comparisons.
