# Supplementary figures and images for: Dietary Macronutrient Intake May Influence the Effects of TCF7L2 rs7901695 Genetic Variants on Glucose Homeostasis and Obesity-Related Parameters: A Cross-Sectional Population-Based Study
Source: Nutrients. 2021 Jun 4;13(6):1936. doi: 10.3390/nu13061936 (PMC8230266; doi:10.3390/nu13061936)

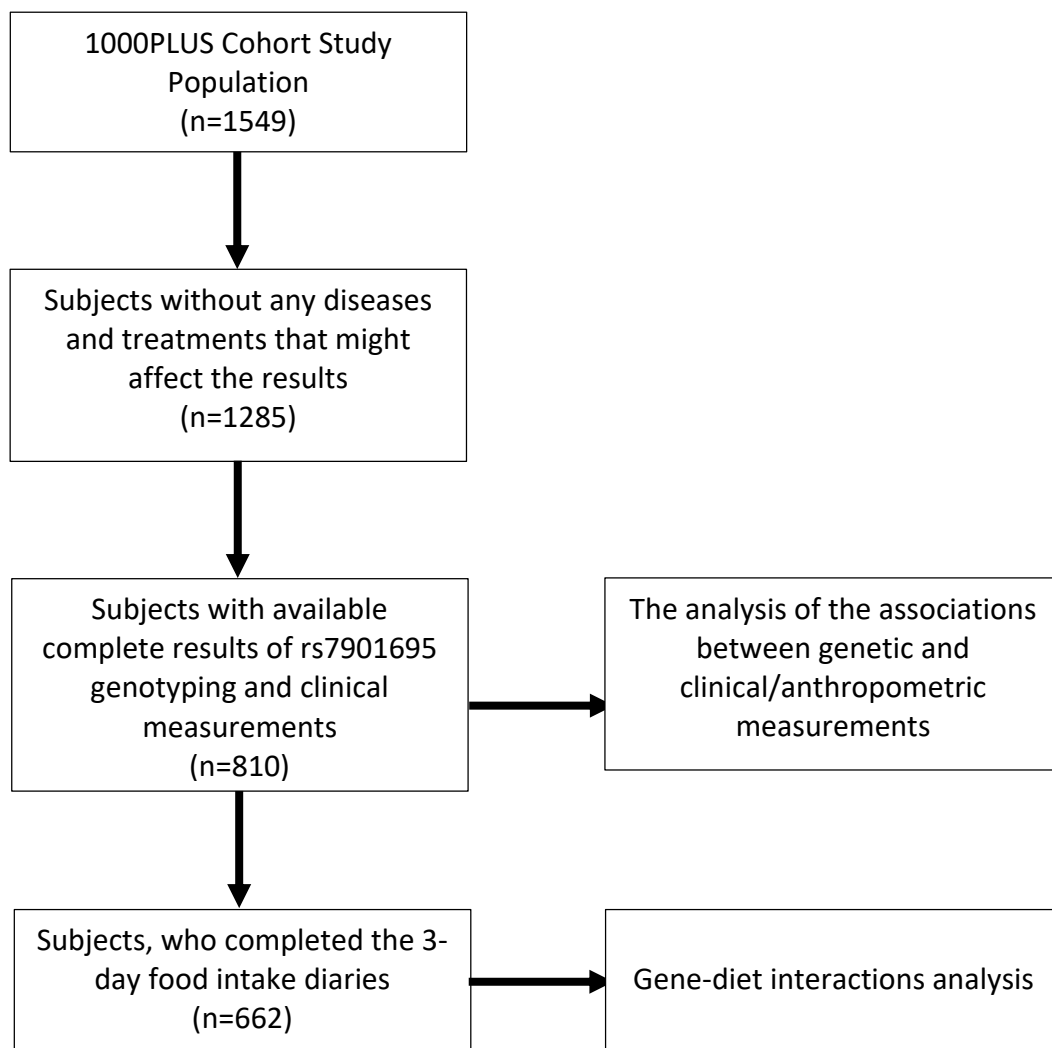

**Supplementary Figure S1.** Study flowchart diagram.

Supplement: Supplementary file 1 [file nutrients-13-01936-s001.zip › Supplementary Figure S1. Flow-chart diagram.pdf]
